# Immunohistochemical expression of EGFR in oral leukoplakia: Association with clinicopathological features and cellular proliferation

**DOI:** 10.4317/medoral.17950

**Published:** 2012-02-09

**Authors:** Daniela C. Ribeiro, Frederico O. Gleber-Netto, Sílvia F. Sousa, Vanessa F. Bernardes, Mauro H.N. Guimarães-Abreu, Maria C.F. Aguiar

**Affiliations:** 1 DDS, MS, Department of Oral Pathology and Surgery, School of Dentistry, Universidade Federal de Minas Gerais; 2 DDS, Department of Oral Pathology and Surgery, School of Dentistry, Universidade Federal de Minas Gerais; 3 DDS, PhD, Department of Oral Pathology, School of Dentistry, Universidade Federal de Minas Gerais; 4 DDS, PhD. Department of Community and Preventive Dentistry, School of Dentistry, Universidade Federal de Minas Gerais

## Abstract

Objectives: to investigate the immunoexpression of epidermal growth factor receptor (EGFR) in a sample of oral leukoplakias (OL) and to determine the receptor’s association with dysplasia, tobacco consumption, lesion site, and proliferation rate. Although EGFR should be overexpressed in some oral leukoplakias, the factors that may interfere with this expression and the influence of this receptor on epithelial proliferation have yet to be investigated. Study Design: Samples of oral leukoplakias (48) and of normal oral epithelium (10) were immunohistologically examined for expression of EGFR. Immunohistochemistry for Ki-67, and p27 were also performed in leukoplakias. EGFR expression was associated with clinical and pathological features. Results: EGFR was positive in 62.5% of the leukoplakias and 50% of normal oral epithelium. The number of EGFR positive OL located in high-risk sites was significantly higher than EGFR positive OL located in low-risk sites. Most of the p27 negative leukoplakias were EGFR positive, and the p27 index in the parabasal layer was diminished in the presence of dysplasia. Positivity for EGFR was not associated with dysplasia, tobacco exposure, or Ki-67. Conclusion: EGFR is expressed in leukoplakia regardless of dysplasia, but EGFR positivity should be more frequent in lesions sited in areas of high cancer risk. The association between EGFR and p27 may represent an important mechanism in the control of cellular proliferation and malignant progression of oral epithelium and therefore warrants further investigation.

** Key words:**Oral leukoplakia, EGFR, p27, Ki-67, epithelial dysplasia.

## Introduction

Leukoplakia is a potentially malignant disorder of the oral mucosa that is defined as a white patch or plaque of questionable risk that cannot be characterized clinically or pathologically as any other known disease (1) Upon biopsy, some leukoplakias may exhibit epithelial dysplasia that is likely associated with progression to cancer (2,3). Other features of leukoplakias, such as smo-king (4,5) and location on the floor of the mouth and/or on the tongue (2,6), have also been associated with an increased risk of malignant transformation.

Several studies have attempted to identify biomarkers that may be useful in predicting malignant transformation (7). The epidermal growth factor receptor (EGFR) is a member of a family of tyrosine kinase receptors that are overexpressed in several types of cancers, including the oral squamous cell carcinoma (OSCC) (8-10). There is substantial evidence that high expression of EGFR is correlated with advanced tumor stages, metastases, and poor clinical outcomes (11). Previous studies have also indicated that EGFR upregulation may be a useful marker for identifying individuals at risk of OSCC development (12-15) However, clinicopathological features of lesions that may interfere with EGFR expression in potentially malignant disorders have yet to be investigated.

The exact mechanisms involved in the control of cellular proliferation through the EGFR pathway are not fully understood. Recently, preclinical studies examining EGFR have shown that tyrosine kinase inhibitors block EGFR tyrosine kinase activity, resulting in inhibition of cell proliferation and upregulation of p27 in OSCC cells (16-18).

The expression of EGFR in leukoplakias is likely altered by clinicopathological features and may modulate proliferation indexes. The present study evaluated the immunoexpression of EGFR in a sample of oral leukoplakia and its association with dysplasia, tobacco consumption, lesion site, and proliferation rates.

## Material and Methods

Sample collection

The study protocol was approved by the Ethics Committee of Universidade Federal de Minas Gerais (ETIC 48/08). Samples with clinical diagnosis of oral leukoplakias (OL) and normal oral epithelium (NOE) were gathered from the files of the Oral Pathology Service, Universidade Federal de Minas Gerais (UFMG), Brazil. Clinical records of each case were evaluated, and tobacco use was investigated. Sections of formalin-fixed, paraffin-embedded incisional biopsy specimens of the OL were evaluated by hema-toxilin-eosin (HE) staining. Forty-eight OL were selected, separated according to site (high and low-risk) and classified histopathologically in two groups according to epithelial dysplasia (absence or presence) following the WHO recommendation (3). The tongue and oral floor were considered high-risk sites, whereas all other intra-oral sites were considered low-risk (2,6). Cases located on the lip were not included in our study. Ten NOE, from different oral sites (low and high risk) were added for comparative purposes.

Immunohistochemistry (IHC)

IHC reactions for detection of EGFR, Ki-67, and p27 antigens were performed using 31G7 monoclonal antibody clones (Zymed Laboratories Inc., San Francisco, CA, UK), MIB-1 (Dako, Carpinteria, USA), and SX5368 (Dako, Carpinteria, USA), respectively. Briefly, 4µm sections were dewaxed in xylene and hydrated with graded ethanol. Blocking of endogenous peroxidase and avidin-biotin activity was performed. Different protocols of antigen retrieval were employed. The antigen retrieval for EGFR was performed with pepsin 10% at 37°C. For Ki-67 and p27, the sections were placed in a steamer containing 10µm citric acid (pH 6.0) for 20 min and Tris/EDTA buffer (pH 8.0) for 20 min, respectively. The primary antibodies for EGFR and Ki-67 were incubated for 18 H at room temperature and diluted 1:100. The antibody for p27 was incubated for 18 H at 4°C and diluted 1:150. All dilutions were in 1% bovine serum albumin.

After rinsing in Tri-HCl buffer, sections were incubated for 30 minutes at room temperature with biotinylated multilink swine anti-goat, mouse, and rabbit immunoglobulin (LSAB Kit, DaKo, Carpinteria, CA, USA). The reaction was revealed by applying diaminobenzidine tetrahydrochloride (solid, DMBA). The sections were then counterstained with Mayer`s hematoxylin and mounted in Permount (Fisher Scientific, NJ, USA). Negative controls were obtained by the omission of the primary antibody.

Evaluation of IHC

The immunohistochemical stain was analyzed by a blind and calibrated examiner (RDC) EGFR expression was evaluated by the extent and intensity of EGFR immunolabeling in cell membranes and classified on a four-point scale as follows: 0 (no labeling or labeling <10% of epithelial cells), 1 (weak labeling, >10%), 2 (moderate labeling, >10%), and 3 (intense labeling, >10%). For data analysis, these categories were divided into two groups, 0 (negative; absent or weak labeling 0, 1) and 1 (positive; moderate and intense 2, 3) (12,19).

Nuclear expression of Ki-67 and p27 was determined for each epithelial layer, including the basal layer (positive nuclei just above the basement membrane), the parabasal layer (positive nuclei within two layers above the basement membrane), and the suprabasal layer (positive nuclei in a more upper layer above the parabasal layer). The percentages of nuclei positively staining for Ki-67 and p27 in 500 epithelial cells for each layer were calculated as the labeling index (LI) (20).

Based on Ki-67 and p27 expression levels in the oral epithelium (20-21), a cutoff value of 20% was set. This value was used to analyze the association between EGFR with Ki-67 and p27. To analyze the differences of cell proliferation in the presence and absence of dysplasia, the mean LIs for each layer were utilized.

Statistical procedures:

SPSS (Statistic Package for Social Sciences) 15.0 for Windows (SPSS Inc., Chicago, IL, USA) was used to analyze data. The differences in expression of EGFR were analyzed using the chi-square test and Fisher’s exact test (p < 0.05). T tests and Mann-Whitney tests (p < 0.05) were used to evaluate the differences of mean/median LIs between dysplastic and non-dysplastic groups for Ki-67 and p27 in each layer.

## Results

Of the 48 cases analyzed, 28 were male and 20 were female. Ages ranged from 24 to 78 years of age, with a mean age of 53 years. With regards to anatomic location, 34 were located in a low-risk site and 14 in a high-risk site. Concerning tobacco consumption, 30 patients were smokers, and 18 were non-smokers. Twenty samples were histopathologically classified as hyperkeratosis with dysplasia, and 28 qualified as hyperkeratosis without dysplasia.

**Figure 1:**
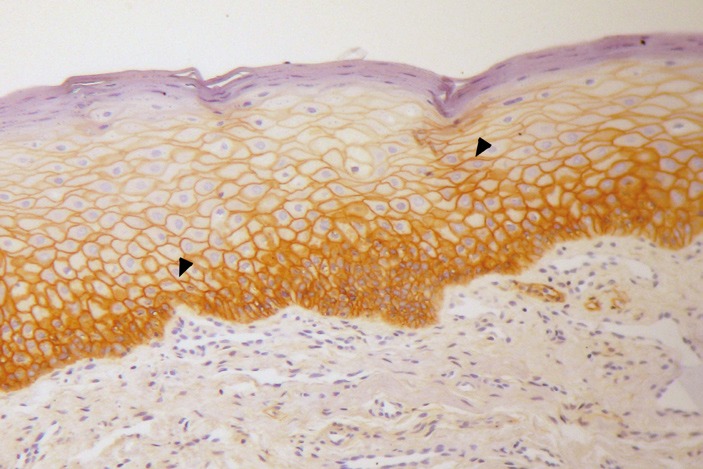
(A) Membranous staining for EGFR in oral leukoplakia (arrowheads). Immunohistochemical staining, original magnification 400x.

EGFR was positive in 30 (62.5%) cases of leukoplakia, and staining was localized to membranes and involved a basal extension to the suprabasal layers (Fig. 1). In NOE, EGFR was positive in 50% of the samples and the staining was restricted mainly to basal and parabasal layers. Although NOE of different sites were included, there was no association between the location of NOE and EGFR immunoexpression.

In oral leukoplakia, EGFR immunoexpression did not show significant variation in the presence or absence of dysplasia or between smokers and non-smokers. There were differences in EGFR expression between leukoplakias in high and low-risk sites, with a higher number of EGFR positive lesions in high-risk than low-risk areas. When all epithelial layers were considered, p27 indexes were also significantly different between EGFR positive and negative leukoplakias. Most of the p27 negative leukoplakias were EGFR positive, but no significant difference was seen for Ki-67 expression with respect to EGFR immunoexpression ( Table 1).

**Table 1:**
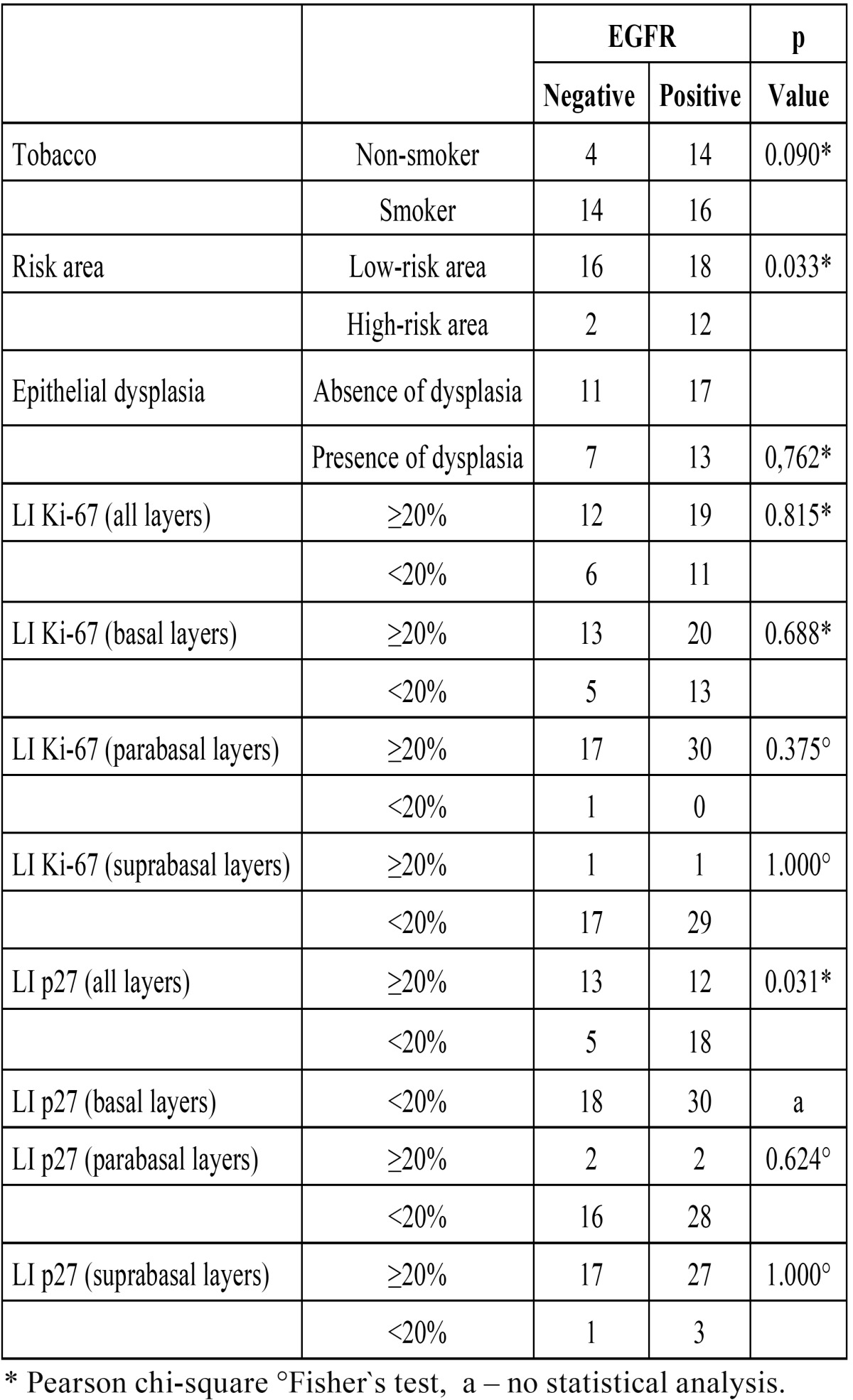
Expression of EGFR according to tobacco smoking, location of lesion, epithelial dysplasia, and cellular proliferation.

Indexes for Ki-67 showed no significant difference between dysplasia and non-dysplasia ( Table 2). In contrast, indices for p27 were significantly lower in the parabasal layer of epithelium with dysplasia than the epithelium without dysplasia ( Table 3).

**Table 2:**
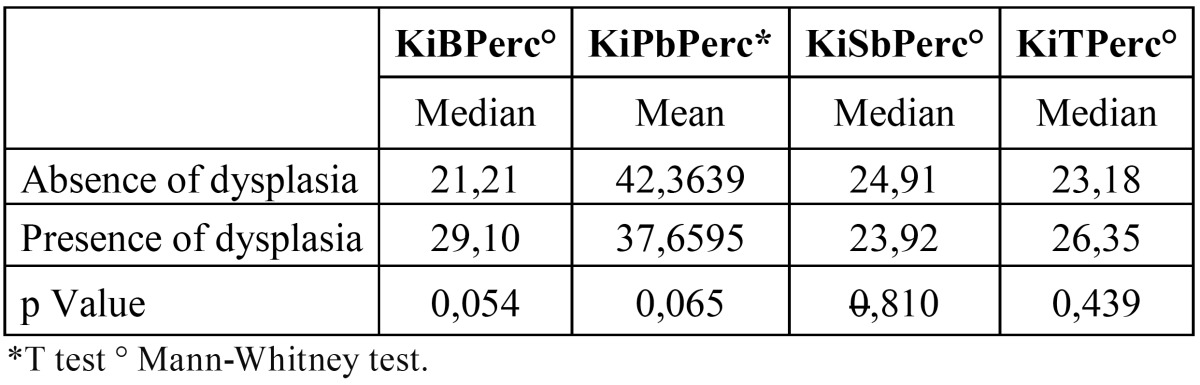
Mean values of Ki-67 labeling indices (LI) in epithelial layers of leukoplakias.

**Table 3:**
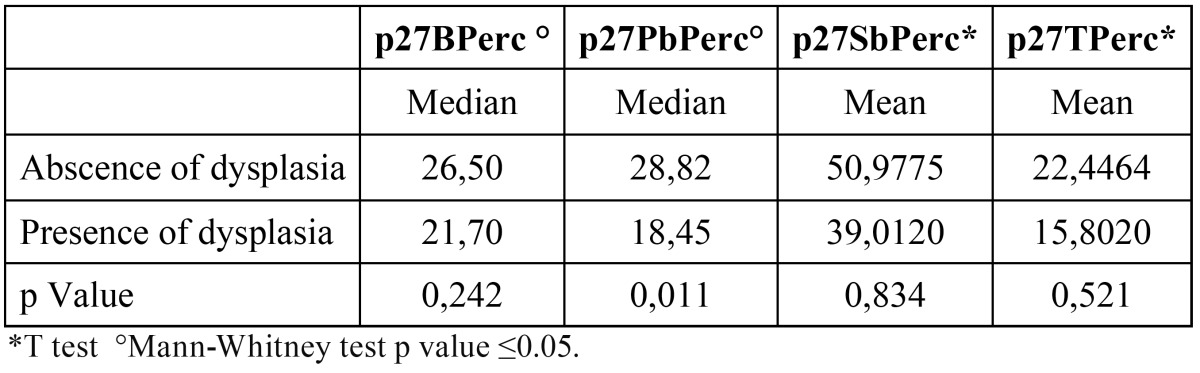
Mean values of p27 labeling indices (LI) in epithelial layers of leukoplakias.

## Discussion

Previous studies have demonstrated increased EGFR expression in oral leukoplakias (13,15). In this study, we evaluated the influence of clinicopathological factors on the immunoexpression of EGFR in a group of oral leukoplakias as well as the association of EGFR and cellular proliferation.

The methods for evaluation of EGFR immunoexpression are variable and include semiquantitative and automated analysis (12,15) with variable indexes and consequently providing controversial results (13,22). Some studies employ methods of evaluation considering the extent and the intensity of the immunostaining, as used in this study (19) while other authors indicate strictly quantitative methods (23).

In leukoplakias it is generally accepted that histopathological characteristics of none or mild dysplasia have a lower risk of malig-nant transformation than moderate or severe dysplasia (2). Some reports describe a dramatic increase in EGFR expression with progression of a dysplastic lesion to cancer without significant differences among various grades of dysplasia (15,23). Consistent with these prior findings, our results did not show differences in EGFR expression between dysplastic and non-dysplastic epithelium.

In the present study, the tongue or floor of the mouth were considered to be high-risk sites for leukoplakia. This statement is consistent with prior studies (1,2). Differences were found in EGFR positivity between leukoplakias from high and low-risk sites. EGFR expression was more frequent in lesions from high-risk than low-risk areas. Another study (6) showed that leukoplakia at high-risk oral sites exhibited more advanced molecular changes (loss of heterozygosity - LOH) than were suggested by the histological findings. The present study reinforces the results of this molecular study.

EGFR expression is associated with smoking history and is significantly upregulated in lung squamous cell carcinoma (24). Tobacco smoking is the strongest independent risk factor for leukoplakia, and studies show that there is a clear dose-dependent relationship for cigarettes (4-5) Interestingly; EGFR expression did not vary in leukoplakias from smokers and non-smokers. Because some reports have shown that tobacco smoking induces several EGFR ligands and subsequently leads to EGFR stimulation (25,26), we recommend that this topic should be further investigated.

Increased cellular proliferation in oral leukoplakia has been reported in prior studies (20-21,27-28) and was confirmed by the present results. Although the Ki-67 LIs value did not correlate with corresponding EGFR positivity or with the presence of dysplasia, the present study confirmed an extended suprabasal expression of Ki-67 in leukoplakia (20). Likewise, although no difference in the Ki-67 LIs associated with dysplasia was found, this index tended to be more pronounced in dysplastic epithelium.

The CDK inhibitor p27 plays an important role in G2 arrest by binding to and inhibiting G1 cyclin (a CDK complex) and negatively regulating progression through the G1 and S phases of the cell cycle. Reduced levels of p27 have been reported in a number of human tumors and have been associated with aggressive histological behavior (29). In the present study, 23 (48%) of the leukoplakias were negative for p27.

A reduction in the expression of p27 in oral leukoplakia in comparison with normal oral epithelium has been described in previous studies (21,29-30) but the role of EGFR in this reduction has not been explored yet. The levels of p27 are mainly regulated via post-transcriptional mechanisms, including ubiquitin-proteosome-mediated proteolysis. The majority of p27 in tumor cells is regulated by ubiquitin degradation (18), and an upregulation of p27 has been associated with downregulation of skp2 via erbB-2 or EGFR inhibition (16,18,31).

In the present sample there was a significant difference between p27 positive and negative leukoplakias. Most of the p27 negative leukoplakias were EGFR positive. Previous studies have shown that gefitinib (ZD1839, Iressa), an EGFR tyrosine kinase inhibitor, causes growth arrest “in vitro” coincident with upregulation of p27 (16-18) Our results are consistent with these findings and suggest that EGFR exerts a role in the control of cell proliferation in OL via p27, but not enhancing Ki-67.

An association between p27 and dysplasia was also observed in the parabasal layer of epithelium. Expression of p27 tended to be reduced in presence of dysplasia, which is in accordance with previous findings (21,30). This suggests that alterations in p27 expression may precede the invasive stages of oral tumorigenesis and reflect changes in growth control of dysplastic cells.

In conclusion EGFR expression is frequent in oral leukoplakia, especially in those lesions located on floor of the mouth and on the tongue. The association of p27 with EGFR warrants additional investigation, and, in the future, may allow for new treatment options for severe leukoplakias through EGFR inhibitors.
